# GECOP-MMC: phase IV randomized clinical trial to evaluate the efficacy of hyperthermic intraperitoneal chemotherapy (HIPEC) with mytomicin-C after complete surgical cytoreduction in patients with colon cancer peritoneal metastases

**DOI:** 10.1186/s12885-022-09572-7

**Published:** 2022-05-12

**Authors:** Fernando Pereira, Angel Serrano, Israel Manzanedo, Estibalitz Pérez-Viejo, Santiago González-Moreno, Luis González-Bayón, Alvaro Arjona-Sánchez, Juan Torres, Isabel Ramos, Maria E. Barrios, Pedro Cascales, Rafael Morales, Enrique Boldó, Alfonso García-Fadrique, Xabier Arteaga, Alberto Gutierrez-Calvo, Susana Sánchez-García, Enrique Asensio, Cesar P. Ramírez, Manuel Artiles, Javier Vaqué, Pedro A. Parra, Pedro Villarejo, Cristóbal Muñoz-Casares, Estrella Turienzo, Alicia Calero, Isabel Jaén Torrejimeno, Isabel Prieto, Julio Galindo, Vicente Borrego, Manuel E. Marcello, Cristina Rihuete, Joaquin Carrasco, Luis Gomez-Quiles

**Affiliations:** 1 Hospital Universitario de Fuenlabrada, Camino del Molino 2, Fuenlabrada, 28942 Madrid, Spain; 2 MD Anderson Cancer Center, Calle de Arturo Soria 270, 28033 Madrid, Spain; 3 Hospital General Universitario Gregorio Marañón, C/ Doctor Esquerdo, 46 -, 28007 Madrid, Spain; 4 Hospital Universitario Reina Sofía, Avda. Menendez Pidal s/n, 14004 Córdoba, Spain; 5 Hospital Universitario Torrecárdenas, Calle Hermandad de Donantes de Sangre s/n, 04009 Almería, Spain; 6 Hospital Sant Joan Despi Moises Broggi, Carrer de Jacint Verdaguer, 90, 08970 Sant Joan Despí, Barcelona, Spain; 7 Hospital Clinico Universitario de Valencia, Avenida de Blasco Ibañez, 17, 46010 Valencia, Spain; 8 Hospital Universitario Virgen de la Arrixaca, Ctra. Madrid-Cartagena, s/n, 30120, El Palmar, Murcia, Spain; 9 Hospital Universitario Son Espases, Carretera de Valldemossa, 79. 07210 Palma, Mallorca, Spain; 10 Consorcio Hospitalario Provincial De Castellón, Avenida del Doctor Clarà 19, 12006 Castellón de la Plana, Spain; 11 Instituto Valenciano de Oncología, C/ Prof. Beltrán Báguena, 8 -, 46009 Valencia, Spain; 12 Hospital Universitario Donostia, Begiristain Doktorea Pasealekua 109, 20014 Donostia, Gipuzkoa Spain; 13 Hospital Universitario Principe de Asturias, Carretera Alcalá-Meco, s/n - 28805 Alcalá de Henares, Madrid, Spain; 14 Hospital General Universitario de Ciudad Real, C/ Obispo Rafael Torija s/n - Pol. Larache, 13005 Ciudad Real, Spain; 15 Hospital Universitario Río Hortega, Calle Dulzaina, 2, 47012 Valladolid, Spain; 16 Hospital Quirónsalud Málaga, Avenida de Imperio Argentina, 1, 29004 Málaga, Spain; 17 Hospital Universitario de Gran Canaria Doctor Negrín, Barranco de la Ballena, 0, 35010 Las Palmas de Gran Canaria, Spain; 18 Hospital Universitario y Politécnico La Fe, Avenida Fernando Abril Martorell, 106, 46026 Valencia, Spain; 19 Hospital General Universitario Reina Sofía, Avda. Intendente Jorge Palacios, 1, 30003 Murcia, Spain; 20 Fundación Jiménez Díaz, Avda. Reyes Católicos 2, 28040 Madrid, Spain; 21 Hospital Universitario Virgen del Rocío, Avda de Manuel Siurot s/n, 41013 Sevilla, Spain; 22 Hospital Universitario Central de Asturias, Avenida de Roma, 0, 33011 Oviedo, Spain; 23 Hospital General Universitario de Elche, Camí de la Almazara, 11, 03203 Elche, Alicante, Spain; 24 Complejo Hospitalario Universitario de Badajoz, Av. de Elvas, s/n, 6080 Badajoz, Spain; 25 Hospital Universitario La Paz, Paseo de la Castellana 261, 28046 Madrid, Spain; 26 Hospital Universitario Ramón y Cajal, Ctra. de Colmenar Viejo km. 9,100, 28034 Madrid, Spain; 27 Hospital Clínico Universitario “Lozano Blesa”, Avda. San Juan Bosco, 15, 50009 Zaragoza, Spain; 28 Hospital Universitario Fundación Alcorcón, Calle de Budapest, 1, 28922 Alcorcón, Madrid, Spain; 29 Hospital Universitario Infanta Elena, Avenida de los Reyes Católicos 21, 28340 Valdemoro, Madrid, Spain; 30 Hospital Regional Universitario de Málaga, Avda. Carlos Haya 82-88, 29010 Málaga, Spain; 31 Hospital General Universitario De Castellón, Avenida de Benicassim, 128, 12004 Castellón, Spain

**Keywords:** HIPEC, Peritoneal carcinomatosis, Peritoneal metastases, Colon cancer

## Abstract

**Background:**

The French PRODIGE 7 trial, published on January 2021, has raised doubts about the specific survival benefit provided by HIPEC with oxaliplatin 460 mg/m^2^ (30 minutes) for the treatment of peritoneal metastases from colorectal cancer. However, several methodological flaws have been identified in PRODIGE 7, specially the HIPEC protocol or the choice of overall survival as the main endpoint, so its results have not been assumed as definitive, emphasizing the need for further research on HIPEC. It seems that the HIPEC protocol with high-dose mytomicin-C (35 mg/m^2^) is the preferred regime to evaluate in future clinical studies.

**Methods:**

GECOP-MMC is a prospective, open-label, randomized, multicenter phase IV clinical trial that aims to evaluate the effectiveness of HIPEC with high-dose mytomicin-C in preventing the development of peritoneal recurrence in patients with limited peritoneal metastasis from colon cancer (not rectal), after complete surgical cytoreduction. This study will be performed in 31 Spanish HIPEC centres, starting in March 2022. Additional international recruiting centres are under consideration. Two hundred sixteen patients with PCI ≤ 20, in which complete cytoreduction (CCS 0) has been obtained, will be randomized intraoperatively to arm 1 (with HIPEC) or arm 2 (without HIPEC). We will stratified randomization by surgical PCI (1–10; 11–15; 16–20). Patients in both arms will be treated with personalized systemic chemotherapy. Primary endpoint is peritoneal recurrence-free survival at 3 years. An ancillary study will evaluate the correlation between surgical and pathological PCI, comparing their respective prognostic values.

**Discussion:**

HIPEC with high-dose mytomicin-C, in patients with limited (PCI ≤ 20) and completely resected (CCS 0) peritoneal metastases, is assumed to reduce the expected risk of peritoneal recurrence from 50 to 30% at 3 years.

**Trial registration:**

EudraCT number: 2019–004679-37; Clinicaltrials.gov: NCT05250648 (registration date 02/22/2022, ).

## Background

Colorectal cancer (CRC) is the most frequent malignant neoplasm in Spain, with 41,441 new cases each year. One-third present with metastases at diagnosis, and another third develop metastases after receiving a presumably curative treatment. Peritoneal spread occurs in approximately 20% of these patients, and in around 8% it is exclusively peritoneal [1], either synchronous or metachronously. Peritoneal metastases (PM) are even more frequent than lung metastases [2, 3].

Until the last decades of the twentieth century, PM were considered a terminal stage of the disease, with a very short life expectancy [4]. The standard treatment was based only in palliative systemic chemotherapy (SCT), considering surgery exclusively for palliative purposes. Although the median overall survival (OS) has increased notably (from 6 to 12–16 months) with contemporary SCT (based on oxaliplatin or irinotecan), it is clearly unfavorable compared to patients treated with the same STC for exclusively hepatic (19.1 months) or pulmonary metastases (24.6 months) [5], and prolonged survival is anecdotal.

The introduction of cytoreductive surgery (CRS) + Hyperthermic IntraPEritoneal Chemotherapy (HIPEC) from the 1990s, but especially after 2000, has obtained unprecedented results in these patients, unattainable exclusively with the current SCT. CRS + HIPEC in patients with limited PM increases survival to 40 months if CRS is complete [6, 7], with a 16% chance of cure [8]. These results are similar to those obtained in the resection of liver metastases [9]. After the expansion of these procedures, the Peritoneal Cancer Index (PCI) has become the most widely used scoring system to estimate the volume of peritoneal disease [10], in the same way as the Completeness of Cytoreduction Score (CCS) to assess the size of residual disease after cytoreduction [11].

It is true that the best current survival data in patients with isolated PM treated exclusively with SCT is around 16 months, but these are unselected patients in whom the volume of peritoneal disease is unknown due to imaging test limitations for its evaluation. The high OS obtained with CRS + HIPEC in multiple series is observed in patients with limited disease. However, no one doubts that these figures are unattainable exclusively with current SCT.

Despite these facts, for many years the results of CRS + HIPEC have generated a certain reluctance because there were no level I scientific evidence studies to support them. This is in contrast to what happened with the resection of liver metastases from CRC, fully accepted in the oncosurgical community due to the accumulated information, despite not being supported by any randomized clinical trial (RCT) [12]. Nevertheless, CRS + HIPEC was gradually accepted for selected patients with PM from CRC, that today is the most frequent indication for CRS + HIPEC, coming to appear in multiple treatment guidelines, and even coming to be considered an standard therapeutic option in various countries [13–15].

The referred results were always achieved including both parts of the treatment, i.e. both CRS and HIPEC, but the role of HIPEC as a necessary component of treatment was not clear, despite its proven experimental basis [16–19], and has not been evaluated separately until very recently. The French PRODIGE 7 study, presented at the 2018 American Society of Clinical Oncology (ASCO) annual meeting [20] and published on January 2021 [21], has raised doubts about the specific survival benefit provided by HIPEC. In this study, there was no benefit in OS with HIPEC (with Oxaliplatin 460 mg/m^2^ for 30 minutes) after resection of PM-CRC, which increased the risk of late complications.

However, the debate has only just begun. PRODIGE 7 is a seminal study, and its researchers should be congratulated. However, it is an isolated study and its information is not considered sufficient, for most experts in peritoneal surface malignancies, to discard HIPEC in this scenario, as several methodological flaws have been identified, specially the HIPEC protocol or the choice of OS as the main endpoint. The PRODIGE 7 researchers themselves suggest that the investigation should be expanded to determine whether any subgroup of patients with PM may benefit from HIPEC, or the potential utility of HIPEC with agents other than oxaliplatin. Given the lack of standardization of HIPEC, PSOGI (Peritoneal Surface Oncology Group International) and RENAPE (*Réseau National de prise en charge des Tumeurs Rares du Péritoine*) groups have launched an international Delphi consensus in November 2021, trying to identify a HIPEC protocol to be evaluated in further clinical trials. It seems that the preferred HIPEC regimen is the one based on high-dose (35 mg/m^2^) mytomicin-C (MMC).

The collaborative network of the Spanish Group of Peritoneal Oncologic Surgery (GECOP: *Grupo Español de Cirugía Oncológica Peritoneal*), under the auspices of the Spanish Society of Surgical Oncology (SEOQ: *Sociedad Española de Oncología Quirúrgica*) [22], provides a great opportunity to deepen the study of the possible benefits of HIPEC. The main objective of our trial is to clarify with greater precision the real role of HIPEC in this setting, trying to correct the methodological flaws detected in the French study.

## Methods/design

### Objectives

The primary aim is to assess whether there are differences in PERITONEAL RECURRENCE in patients with limited-volume colon cancer PM treated with complete surgical resection and systemic chemotherapy, with or without HIPEC with MMC.

Secondary Objectives are:

- Evaluate whether there are differences in global disease recurrence (at any location) between both groups (disease free survival-DFS).
- Assess the toxicity of the treatments and compare the postoperative complications between both groups.
- Determine prognostic factors for peritoneal relapse and recurrence at other sites.
- Compare overall survival between both groups.
- Study of the quality of life in both groups using EORTC (European Organisation for Research and Treatment of Cancer) validated questionnaires.
- Correlation between surgical and pathological PCI, comparing their respective prognostic values (ancillary study).

### Design

**Fig. 1:**
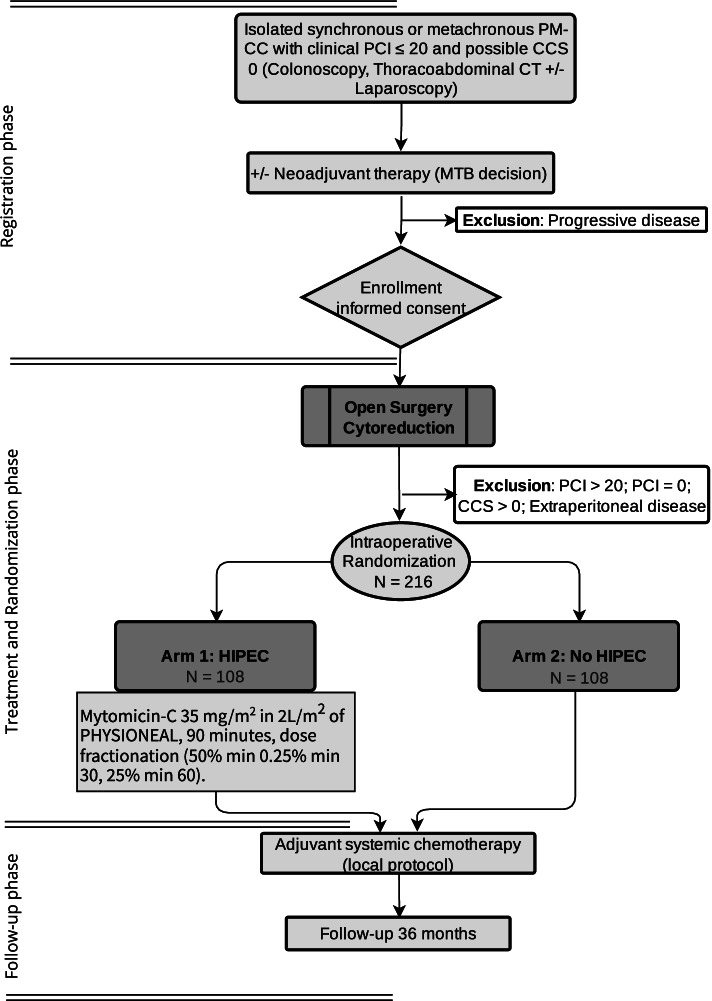
Flow-diagram GECOP-MMC trial. PM-CC: peritoneal metastases from colon cancer; PCI: Peritoneal Cancer Index; CCS: Completeness of cytoreduction score; MTB: multidisciplinary tumour board; HIPEC: hyperthermic intraperitoneal chemotherapy

This will be a multicenter, prospective, open-label, randomized (1:1) phase IV clinical trial in patients undergoing CRS for PM from colon cancer, with (Arm 1) or without (Arm 2) HIPEC with high-dose MMC (Fig. 1). This study will be performed in 31 Spanish HIPEC centres, starting in March 2022. Additional international recruiting centres are under consideration. The protocol adheres to SPIRIT guidelines for reporting clinical trial study protocols.

It is called phase IV since, after the results of PRODIGE-7, both treatments (CRS + HIPEC vs a procedure based exclusively on CRS, eliminating HIPEC as a component of treatment) could be considered standard.

### Study population (eligible patients)

Patients with colon cancer PM who are considered for CRS by a Multidisciplinary Tumour Board (MTB), and can present in various clinical scenarios:

- Synchronous PM: patients with primary colon tumours “in situ” with synchronous peritoneal disease and presumable possibility of complete CRS (Primary Surgery).
- Persistence of synchronous PM: patients with resected primary colon tumours in whom synchronous peritoneal disease is discovered but not completely removed at the time of initial surgery, considering that complete CRS is possible (Rescue Surgery).
- Metachronous PM: patients with resected primary colon tumours that relapse in the peritoneum, in whom a complete CRS seems possible (Secondary Surgery).

The inclusion criteria are:

1. Histologically confirmed colon adenocarcinoma, except signet ring cell carcinomas (those with > 50% of the tumour composed of these cells, which comprise only 1% of all colon adenocarcinomas).
2. Absence of previously treated or current extraperitoneal metastases, including distant lymphadenopathy (retroperitoneal, mediastinal, etc), liver metastases, or lung metastases (ruled out by PET-scan in case of doubt).
3. Synchronous or metachronous peritoneal metastasis of mild to moderate volume, with a PCI ≤ 20 (intraoperative confirmation).
4. Macroscopically complete surgical cytoreduction: CCS = 0 (intraoperative confirmation).
5. Treatment with perioperative SCT, before and/or after the surgical procedure.
6. Age > 18 years.
7. Acceptable anaesthetic/surgical risk: ASA 1–3, ECOG 0–1. No severe alterations in hematological, renal, cardiac, pulmonary or hepatic function (operable patients).
8. Information to the patient and signing of a study-specific informed consent.

And the exclusion criteria are:

1. Peritoneal Carcinomatosis of any other origin, particularly rectal cancer or appendiceal adenocarcinoma, or signet ring cell colon cancer on histology.
2. No intraoperative confirmation of peritoneal disease (PCI 0). Likewise, cases of perianastomotic (local) or lymph node (locoregional) recurrences will be excluded.
3. High volume peritoneal disease with a PCI > 20 (intraoperative confirmation).
4. Concurrent or previously-treated extraperitoneal disease.
5. Disease progression during preoperative SCT, if received.
6. Patients previously treated with HIPEC.
7. History of other cancers (except cutaneous basal cell carcinoma or cervix carcinoma in situ) in the 5 years prior to entry into the study.
8. Patients included in another first-line clinical trial for the studied disease.
9. Pregnancy (or suspicion of it) or lactation period.
10. Emergency surgical intervention for obstruction or perforation of a primary tumour with synchronous PM (although rescue and secondary CRS ± HIPEC after emergency surgery of the primary tumour are acceptable if inclusion criteria are fulfilled)
11. Persons deprived of liberty or under legal or administrative supervision.
12. Inability to understand the nature of the intervention, the risks, benefits, expected evolution and the need to undergo periodic medical examinations, either for geographical, social or psychological reasons.

The exclusion of patients with PM from rectal cancer must be ensured. Likewise, metastasis that are exclusively extraperitoneal, whether perianastomotic (local) or lymphatic (locoregional or distant, including retroperitoneal), must be excluded. Cases with preoperative suspicion of peritoneal disease, in which its extra-peritoneal location is confirmed intraoperatively, should be excluded from randomization.

Patients will be randomized intraoperatively only when it is confirmed that intraoperative PCI is ≤20 and a complete CRS (CCS-0) has been achieved.

Patients previously treated, even radically, for extraperitoneal metastases (liver or lung) will be excluded, since although the main objective of the study is peritoneal recurrence, the inclusion of these cases could predictably affect OS.

There is no upper limit for chronological age in this study. The limit is imposed by the functional situation (ASA, ECOG), so that results can be extrapolated as best as possible to the “real” population.

### Preoperative work-up

The following assessments should be performed to check the inclusion and exclusion criteria:

- Detailed anamnesis, compiling all the past medical-surgical history.
- Determine ASA and ECOG status.
- Diagnostic tests:
  1. Laboratory tests: Complete blood cell count, coagulation, and basic biochemistry with liver profile. Visceral proteins (albumin and prealbumin). Serum tumour markers: CEA and CA 19–9.
  2. Colonoscopy: only patients with colon cancer will be included, i.e., above 15 cm from the anal verge. In every scenario (primary, rescue and secondary surgeries), this issue will fall on the initial diagnostic colonoscopy, to discard rectal cancers. Virtual colonoscopy is recommended to rule out synchronous lesions if complete colonoscopy is not feasible. In rescue and secondary surgeries, a recent colonoscopy is required in the 4 months prior to surgery to rule out metachronous colonic tumours.
  3. Endoscopic biopsies: cases with > 50% of signet ring cells in the biopsy will be excluded. Immunohistochemistry for DNA repair proteins (to assess microsatellite instability) and test for RAS/RAF mutations will be performed.
  4. Preoperative imaging tests. They have a double objective. On one hand, to exclude the presence of extra-abdominal disease and, on the other, to assess the resectability of intra-abdominal disease. For the latter, knowledge of the size and distribution of the peritoneal implants, as well as involvement of the small intestine and its mesentery is of vital importance. Although with its known limitations [23, 24], the reference-imaging test to assess the extent of PM is multidetector CT with oral and intravenous contrast, complemented by Magnetic Resonance if needed for doubtful intraperitoneal findings. PET-CT will be used selectively, mainly to rule out extra-abdominal disease.
    The use of the free internet application PROMISE (PeRitOneal MalIgnancy Stage Evaluation, www.e-promise.org) is strongly recommended for the estimation of radiological PCI. This application offers computer-assistance to produce simple, quick but precise and standardized pre, intra and postoperative reports of the extent of peritoneal metastases, and may help specialized and non-specialized institutions in their current practice, but also facilitate research and multicenter studies on peritoneal surface malignancies.
  5. Staging laparoscopy: given the usual underestimation of the volume of peritoneal disease in radiological studies [23, 24], staging laparoscopy will be used optionally in suitable cases (those without previous and extensive open abdominal surgeries). Laparoscopy can better characterize the peritoneal tumour volume, and, above all, it can help to rule out as far as possible the involvement of areas that may limit the procedure, mainly the small intestine and its mesentery, even knowing that laparoscopy also tends to underestimate the volume of peritoneal disease [25, 26]

All cases will be evaluated individually in the MTB, proposing entry into the study to those who meet all the presurgical requirements. All these patients are metastatic and candidates for perioperative SCT for at least 6 months, although the sequence (pre or postoperative or both) will be individualized in each case. The SCT regimen will be chosen by the medical oncologist according to information on response (if any) to prior SCT, and should be one of the currently accepted regimens for metastatic colorectal cancer. If patients receive preoperative SCT, they should be reassessed after a short course of treatment (3–4 cycles) and surgery should be avoided if there is tumour progression. SCT should be stopped at least 4 weeks before the intervention, or 6 weeks if bevacizumab is included in the neoadjuvant treatment.

Likewise, indication for laparoscopy and sequence of re-evaluation tests following neoadjuvant SCT will be decided in the MTB, in order to make subsequent decisions.

### Recruitment, informed consent, and registration in the trial

Recruitment will be carried out at the outpatient clinic, once the indication of CRS ± HIPEC (PM of apparently limited volume without metastases at other sites) has been established after presenting the case in the MTB, checking that eligible patients meet the presurgical inclusion criteria (although some criteria have to be confirmed during surgery for randomization). At that time, eligible patients will complete the basal health-related quality of life questionnaires; they will sign a specific Informed Consent, and they will be registered in the trial. Women in childbearing age will be informed that they should avoid pregnancy for at least 1–2 years after finishing treatment.

Nevertheless, during surgery patients could be withdrawn from the trial if they do not meet the intraoperative inclusion criteria (confirmation of peritoneal disease, low to moderate volume PM with PCI ≤ 20, absence of extraperitoneal disease, and complete CRS-CCS 0 achieved), and it is not until then when randomization is performed.

### Treatment and randomization

Patients must undergo OPEN SURGERY, through a midline laparotomy, to avoid bias of PCI underestimation if the laparoscopic approach is admitted. The first step is to confirm definitively that peritoneal tumour volume is not excessive (PCI ≤ 20) and that complete cytoreduction is possible without prohibitive sequelae, otherwise the surgical procedure is aborted. Patients with PCI > 20, or those in whom there is no confirmation of peritoneal disease, are withdrawn from the study intraoperatively. Cases with preoperative suspicion of peritoneal disease, in which during surgery it is noted that the disease is not peritoneal but extraperitoneal, should also be rejected from randomization, same as those in which unexpected systemic disease (eg, liver metastases) is discovered. The goal is to resect all visible disease, without residual tumour nodules, so that HIPEC, if used, is effective. Those cases in which a complete CRS (CCS 0) is not achieved are withdrawn intraoperatively from the study.

In all cases, the greater omentum and cecal appendix (target organs) are resected, if they have not been previously excised. In postmenopausal women the adnexa are also removed even if they are not affected.

Once complete CRS is achieved, patients are randomized 1:1 to receive HIPEC with MMC (Arm 1) or not (Arm 2). Intestinal anastomoses can be performed before or after HIPEC if the patient is randomized to the control arm (Arm 1). Randomization will be done through a computer program (computer-generated random numbers) generated by the Clinical Research Organization (CRO), and will be stratified by surgical PCI: 1–10; 11–15; 16–20.

Any HIPEC modality can be used (open, closed or closed with CO_2_). HIPEC will be performed with MMC, at an average intraperitoneal temperature of 42 °C, and a dose of 35 mg/m^2^ in peritoneal dialysis solution (PHYSIONEAL 35 Glucose 1.36% w/v / 13.6 mg/dl, or equivalent) for 90 minutes (with dose fractionation: 50% at minute 0, 25% at 30 minutes, and 25% at 60 minutes of perfusion). The MMC will be reconstituted in 3 syringes with the corresponding fractions. The volume of solution will be 2 L/m^2^, adapting it to the capacity of the individual abdominal cavity.

### Postoperative phase

The most common intraoperative and early postoperative adverse effects (hemoperitoneum, gastrointestinal bleeding, anastomotic leak, digestive fistula, urinary fistula, seroma, surgical site infection, evisceration, urinary infection, atelectasis, pneumonia, renal failure, central catheter infection, thromboembolism, hematological toxicity if HIPEC is administered) are detected during hospital admission, and will be collected in the electronic Case Report Form. They will be classified according to the CTCAE system (Common Terminology Criteria for Adverse Events) v5.0 [27]. Any other deviation from the normal postoperative course (including minor complications such as vomiting, diarrhea, pain, etc.), red blood cells transfusion if it occurs, and length of stay (discharge date) will also be recorded. All complications will be recorded up to the 90th postoperative day, whether those occurred during hospital admission or those that present later, in readmissions (if they occur), or after discharge not requiring readmission, which will be recorded in follow-up visits.

Upon final discharge, patients will be scheduled for the first postoperative visit. They will also be presented again in the MTB for subsequent management, and an appointment in Medical Oncology will be made to complete SCT.

### Follow up

Patients will return to standardized follow-up visits every 4 months for the first two years, and every 6 months during the third year, at the end of which the last trial visit occurs. Subsequently, visits will continue outside the trial according to the protocol of each centre, normally every 6 months during the 4th and 5th year, and annually thereafter until the 10th year. At each revision, contrast-enhanced thoraco-abdomino-pelvic CT scan and regular laboratory blood test with tumour markers (CEA, CA 19.9) will be requested. A complete colonoscopy is recommended at the 1st postoperative year (and later on according to the standard surveillance protocols in CRC). The tests can be ordered before the scheduled dates and/or other complementary tests can be added depending on the clinical situation.

### Quality of life

Given the importance of assessing not only the strict biomedical results of treatments, but also the patient’s personal perception of their impact, patients will complete the health-related quality of life questionnaires of the European Organization for Research and Treatment of Cancer (EORTC) Core 30 (QLQ-C30) and Colorectal Cancer Module (QLQ-CR29). These tests will be completed at the time of recruitment (before randomization), at the end of SCT (average 4 moths), and during follow-up visits at 12 and 24 months. The QLQ-CR29 is a supplementary questionnaire module to be employed in conjunction with the QLQ-C30. In fact, their numbering is consecutive (the last item of QLQ-C30 is number 30, being the first item of QLQ-CR29 number 31). Both have function and symptom scales/single-items. All of the scales and single-item measures range in score from 0 to 100. A high score for the functional scale and functional single-items represents a high level of functioning, whereas a high score for the symptom scales and symptom single-items represents a high level of symptomatology or problems.

### Endpoints of the study

The primary endpoint of the study is Peritoneal Recurrence Free Survival (RFS) at 3-years.

The secondary endpoints are:

- Global recurrence rate (DFS) at 3-years.
- Locoregional and distant recurrence rate (isolated or coincident, with or without simultaneous peritoneal recurrence) at 3-years.
- Postoperative complications using the CTCAE v5.0 adverse event classification system up to the 90th postoperative day, including those related to HIPEC.
- Prognostic factors for peritoneal and global recurrence: synchronous/metachronous PM, perioperative SCT, use of biological agents or immunotherapy, stratified PCI (1–10, 11–15, 16–20), postoperative complications, right/left colon, degree of tumor differentiation, vascular/lymphatic/perineural invasion, RAS/RAF status, microsatellite instability, and degree of peritoneal tumour regression (if applicable).
- Overall survival rate (OS) at 3-years.
- Study of the Quality of Life in both groups using the EORTC questionnaires QLQ-C30 and QLQ-CR29 at the mentioned time spots.

### Ancillary study

Once the pathology report has been obtained, the surgical PCI will be recalculated, corroborating or invalidating the involvement of the scored lesions, thus obtaining the pathological PCI. Correlation between surgical and pathological PCI will be analysed, comparing their respective prognostic values.

### Sample size calculation

Although median OS of patients with limited PM-CRC treated with CRS + HIPEC (40 months) is much higher than that estimated if they received only SCT (16 months), relapse after CRS + HIPEC is very common. It is difficult to ascertain the actual incidence of recurrence in the literature, with very variable published figures. Van Oudheusden et al. in a systematic review on the item [28] point out that the global recurrence after CRS + HIPEC ranges between 22.5–82%, the local one ranging between 6 and 42.5%, the systemic one between 10.4–43%, and the combined (local + systemic) between 5.8–21.5%, with a median time to recurrence of 9–23 months and a 3-year RFS of 14–41.5%. However, it is estimated that only 16% are cured (5-year RFS rate) and therefore the majority (> 80%) of patients relapse, almost all of them in the first two years, and a high percentage (30–50%) do it at the peritoneum (either isolated or with metastases at other sites).

Sample size was calculated with a bilateral hypothesis, a statistical power of 80% and an alpha level of 0.05. In order to verify a reduction in peritoneal recurrence at 3 years of 20% in the group treated with HIPEC (assuming a probability of 3-year peritoneal relapse of 50% in the group without HIPEC and 30% in the group with HIPEC), 103 patients are needed in each arm (206 in total). Corrected for a 5% of possible losses, 108 participants will be needed in each arm, requiring 216 patients.

### Data analysis

Data will be collected in an ad-hoc electronic Case Report Form (eCRF) already designed in REDCap (Research Electronic Data Capture). Descriptive analysis of the data will be carried out. Qualitative variables will be presented by their frequency distribution (proportions) and quantitative variables will be measured by indicators of central tendency (mean or median) and dispersion (standard deviation or interquartile range respectively).

Hypothesis contrast tests will be performed, with comparison of proportions when both variables are qualitative (Chi square test for normal distribution, Fisher exact test for non-normal distribution) and comparisons of means for independent samples when one of them is quantitative (Student t-test if normal distribution or Mann-Whitney U-test for variables that do not comply with normality).

Survival will be estimated with the Kaplan-Meier method and survival curves will be compared using the Log-Rank test to analyse the effect of the different factors that can influence survival.

A Cox proportional hazard model will be performed to evaluate the effect of representative covariates on RFS. For this, those that are significant in the univariate analysis (*p* < 0.2) or clinically relevant will be included.

A *p* value < 0.05 will be considered statistically significant.

Study results will be analyzed by intention to treat. A second analysis will be conducted according to the treatment actually administered (per protocol analysis). After the recruitment of half of the patients, an interim data analysis of toxicity will be performed, in which severe adverse events with their accountability will be described. The final analysis will be carried out in two phases, one on the morbidity and mortality results at the end of recruitment, and another one at the end of the study with the definitive data on peritoneal disease control and survival. The coordinating investigator will be in charge of writing and publishing the results, complying with international CONSORT (Consolidated Standards of Reporting Trials) recommendations, and with the collaboration of the entire research team.

Stopping guidelines: Study participation by individual sites or the entire study may be prematurely terminated, if in the opinion of the Coordination Centre there is sufficient reasonable cause. Any investigator who wants to discontinue his/her participation to the study must immediately inform the Coordination Centre of this decision. Written notification documenting the reason for study termination will be provided to the Coordinating Investigator by the terminating party.

### Ethics, Regulatory & Legal Considerations

The study is carried out under the ethical principles that appear in the revised version of the Declaration of Helsinki (Fortaleza, Brazil 2013), the Convention on Human Rights and Biomedicine (Oviedo 1997), the Law 41/2002 on Patient Autonomy, the ICH (International Conference on Harmonization) guidelines on “Standards of Good Clinical Practice” (CPMP/ICH/135/95), and complying with the current European (EU Regulation 536/2014) and Spanish (Royal Decree 1090/2015) legislation on clinical trials.

Similarly, the research team undertakes to ensure the privacy of patient data in accordance with the new legislation in the European Union on personal data, specifically the General Data Protection Regulation (GDPR) of the European Parliament and of the Council of April 27, 2016 (2016/679; https://www.boe.es/doue/2016/119/L00001-00088.pdf), fully applicable throughout the European Union since May 25, 2018.

The trial (protocol version 11) obtained authorization both from the Clinical Research and Ethics Committee (CREC) of the IdiPAZ (Hospital La Paz Research Institute) on April 7, 2021 (Code 5814), and from the Spanish Agency for Medicines and Sanitary Products (AEMPS) on April 21, 2021. The mentioned CREC also approved an amendment (protocol version 12) on February 10, 2022. Any other major changes in the study protocol must be documented in protocol amendments that must be submitted to and approved by the CREC, prior to their implementation.

The contracts between the Coordination Centre and each of the participating centres are in process since mid-February 2021 and most of them completed, which is an essential condition to start recruitment.

### Monitoring/audit/safety

The Clinical Research and Clinical Trials Unit (UICEC: *Unidad de Investigación Clínica y Ensayos Clínicos*) of Hospital Universitario La Paz (UICEC-HULP), independent from the Sponsor/Coordinating Centre (*Hospital Universitario Fuenlabrada*) and with no competing interests, will carry out the monitoring and pharmacovigilance of the trial, acting as CRO. Representatives of the CRO will visit the investigators from each centre periodically to monitor the progress of the study in accordance with Good Clinical Practice regulations. It is the responsibility the Investigators to be present or available for consultation during such scheduled monitoring visits. During these routine visits, all data pertaining to a patient’s participation in this clinical investigation must be available to the monitor. On-site review of the eCRF for completeness and clarity, cross checking with documental sources, and reconciliation and clarification of administrative matters will be performed.

In addition, a representative of the regulatory agency (AEMPS) may choose to inspect a study centre at any time prior to, during, or after completion of the clinical study. A Coordination Centre representative or designee will be available to assist in the preparation for such an inspection. All pertinent study data should be made available as requested to the regulatory authority for verification, audit, or inspection purposes.

All adverse effects that occur during the intervention, in the postoperative period (both the most serious and the mildest), or in the course of patient follow-up will be collected in the clinical records. The eCRF will record all adverse events that occur in the postoperative phase, up to the 90th postoperative day. All Serious Adverse Events (SAE), regardless of treatment group or suspected relationship with study treatment, should be reported to the Responsible of Pharmacovigilance at UICEC-HULP within 24 hours of knowledge of the episode.

### Patient participation and withdrawal

Participation of patients is completely voluntary, and they have the right to withdraw from the trial at any time without causing them any harm. If a patient withdraws from the study, the reason will be recorded. Among these reasons it is worth highlighting:

- At the request of the patient, without causing him/her any harm.
- During surgery, if the intraoperative inclusion criteria are not met (PCI ≤ 20 and CCS 0).
- Loss to follow-up.
- Major protocol deviation.
- If the researchers consider it appropriate from a clinical point of view.

When a patient decides to withdraw from the study, he/she should always be contacted in order to obtain information about the reason for withdrawal, and requested to return for a follow up visit, if applicable, and posterior follow-up regarding any potential adverse even and subject outcome, if possible. A patient is considered lost to follow-up if no information has been obtained when the last patient has completed the clinical phase of the study. During this time there must be documented attempts to contact the patient either by phone or letter.

## Discussion

### Prodige 7 consequences and potential flaws

In PRODIGE-7, 265 patients with exclusively peritoneal metastases (without other metastatic locations) and PCI ≤25 were randomized, during the surgical procedure and after achieving a complete or almost complete CRS (residual tumor < 1 mm), to receive HIPEC with oxaliplatin 460 mg/m^2^ for 30 minutes or not. The study took place in 17 French centres, from February 2008 to January 2014. Its results are well known, with no difference in OS (main endpoint of the study) or RFS between both groups, and with an increase in late severe morbidity in the HIPEC group.

With these results, a great debate has been opened on the role of HIPEC in the treatment of PM-CRC. However, the topic is now hotter than ever, as several potential flaws has been identified in PRODIGE 7. Despite the merit of the study, and as has happened with other RCTs, opinions are emerging that downplay its initial impact [29–32]. Some experts already anticipated a negative result for PRODIGE 7 based on its design [33]. There are several arguments to explain these negative results and propose new alternatives to continue evaluating HIPEC in PM-CRC:

- An important limitation of PRODIGE 7 is an overestimation of the effect of HIPEC on OS, calculating that it would produce an 18-month increase in median OS (from 30 to 48 months). The increase in OS recommended in an RCT for the authorization of a systemic treatment is always much more modest, and would not be greater than approximately 5 months for an RCT like PRODIGE 7 [34, 35], although the demonstration of a lower benefit would have required a much higher sample size.
- Another conflicting point is the choice of OS as the main endpoint. HIPEC is a locoregional treatment that can reduce peritoneal recurrence, and only a minority of treated patients are cured [8]. OS of these patients is influenced by the systemic treatment that all of them receive, both perioperatively and at relapse, and its choice as main endpoint distorts the effect of HIPEC itself. This is comparable to the use of neoadjuvant radiochemotherapy in locally-advanced rectal cancer, which reduces local recurrence and is accepted as standard treatment, although it has not been shown to increase OS. Furthermore, the peritoneal recurrences of 12% of the patients in the group without HIPEC were treated with a new CRS + HIPEC (allowing cross-over), which further complicates the analysis of OS. To assess the effect of HIPEC reliably, it would have been more logical to choose the peritoneal recurrence-free survival as the main endpoint. In fact, in PRODIGE 7 the 1-year RFS was 59% in Group A (HIPEC) and 46.1% in Group B (no HIPEC), suggesting that HIPEC delays relapse. Therefore, these results do not exclude (but rather support) a potential benefit of HIPEC in the locoregional control of the disease.
- The choice of the HIPEC protocol used in PRODIGE 7 is highly questionable. Various experts have suggested that the reduced time of exposure to the drug is insufficient to achieve the desired effect [36]. Furthermore, IV 5-FU is used as only a bolus dose, anticipating a low response rate to oxaliplatin (continuous 48 h 5-FU infusion is today an integral part of oxaliplatin treatment); low duration of heat exposition is unlikely to have any major effect by itself; no study demonstrated a potentiation of oxaliplatin cytotoxicity by heat; and the high rate of postoperative complications may have a negative impact on survival of these metastatic patients.
- It seems increasingly clear that oxaliplatin-based preoperative SCT, commonly used in PRODIGE 7, can induce a certain degree of tumour resistance, worsening the efficacy of subsequent intraperitoneal oxaliplatin [37].
- PRODIGE 7 included patients with PCI up to 25, when today it is known that cases with PCI > 20 do not benefit from CRS + HIPEC. It is true that this information has been mainly accepted since the publication of the French registry in 2010 [38], and PRODIGE 7 began recruiting in February 2008. Furthermore, PCI was determined after neoadjuvant SCT in the majority of patients, and therefore, assuming a high response rate, it is likely that the initial PCI in some of these patients was even higher, and perhaps not curable with CRS + HIPEC. However, although a reliable assessment of PCI prior to rather than after neoadjuvant SCT would be the best option, this does not seem to be possible given the limited capacity of imaging tests [23, 24], or even laparoscopic staging [25, 26], to reliably diagnose the volume of peritoneal disease, which is usually understaged. It is also unknown whether PRODIGE 7 included patients with PCI = 0, since it only identifies a global group with PCI < 11.
- PRODIGE 7 also included cases with incomplete cytoreductions (10% of CRS were R2, although with tumour residue < 1 mm), in which a lower benefit of HIPEC is assumed. In addition, more than 10% of the included cases corresponded to rectal cancers, whose biological behaviour is very different from colon cancer, in such a way that several authors even question the indication of HIPEC in this scenario [39].

What is truly striking in PRODIGE 7 is the unexpected OS obtained in the Surgery Only-Group, with a median OS greater than 40 months. Therefore, one of the fundamental conclusions of the study is that patients with PM-CRC should continue to be referred to expert centres to guarantee an adequate selection, a high-quality surgery, and consequently, the best survival outcomes [40]. In addition, this will continue to expand investigation on HIPEC, to determine its potential utility with agents other than oxaliplatin, or whether any subgroup of patients with PM may benefit from HIPEC, as suggested by the PRODIGE 7 researchers themselves. In fact, a post-hoc exploratory analysis of PRODIGE 7 suggests that HIPEC may have a role in the subgroup of patients with PCI 11–15.

### Interest of a new clinical trial evaluating the use of HIPEC

Therefore, the debate on the specific role of HIPEC in PM-CRC has only just begun, and despite the results of PRODIGE 7, HIPEC is still considered a recommended option for these patients worldwide, even in French centres [41]. After PRODIGE 7, all patients receiving CRS + HIPEC for PM-CRC should ideally be included in prospective and translational clinical trials. In Spain, activity in peritoneal cancer surgery is very high. Several groups have gained extensive experience in the last two decades, and various clinical trials have already been designed on the use of HIPEC in other indications. The collaborative network of the Spanish Group of Peritoneal Oncologic Surgery (GECOP) [22], provides a great opportunity to deepen the study of the possible benefits of HIPEC for PM-CRC.

The main objective of our RCT is to clarify with greater precision the real role of HIPEC in this setting, trying to correct the methodological flaws detected in the French study. In order to achieve this goal the drug used in HIPEC will be changed (MMC instead of oxaliplatin), the perfusion time increased (from 30 to 90 minutes), rectal cancers will be excluded (only colon cancers will be included), cases with high peritoneal extension (PCI > 20) or those in which the existence of peritoneal disease is not histologically proven (PCI = 0) will be avoided despite the preoperative suspicion, those cases in which complete CRS is not achieved (CCS 0) will be excluded, and we will change the primary endpoint to be the peritoneal RFS instead of OS.

In addition, considering the significant difference in the median OS in the PRODIGE 7 subgroup of patients with PCI 11–15 in favour of the HIPEC-group (41.6 vs 32.7 months, *p* = 0.0209), which suggests that HIPEC may have a role in this subgroup, we will carry out a stratification of randomization according to PCI, trying to clarify this point.

Furthermore, this clinical trial will include an assessment of quality of life from the perspective of the patients themselves (patient-reported outcomes), also absent in the results of PRODIGE 7.

Finally, surgical PCI (the one reported in PRODIGE 7, which is the one commonly used) strongly differs from pathological PCI [42, 43], that may provide a more accurate evaluation of the peritoneal disease extent, although its prognostic importance has yet to be established. Therefore, we will carry out an ancillary study to compare the prognostic values of both PCIs.

It will be a multicentre study, to which all the GECOP-accredited Spanish Peritoneal Surface Oncology Units have been invited. The trial could even be opened to institutions from other countries, provided regulatory issues can be sorted out.

### Choice of the HIPEC protocol

Although the Elias protocol [44] was widely adopted after an initial publication with promising results [45], it is clear that after PRODIGE 7 this HIPEC protocol should be changed to continue delving into the possible beneficial effects of HIPEC. Several suggestions have been made to improve the HIPEC with oxaliplatin [46]: combining oxaliplatin with irinotecan, increasing the dose of 5FU IV to potentiate the effect of intraperitoneal oxaliplatin, reducing the dose of oxaliplatin (to 200 mg/m^2^) with four times longer perfusion time (120 minutes), or limiting HIPEC with oxaliplatin to those patients who have not received preoperative FOLFOX.

Another alternative would be to go back to MMC-based HIPEC, alone or in combination with other cytostatics, which seems to be the preferred option, since it may be especially important in patients previously treated with neoadjuvant FOLFOX, a widely used regimen. PRODIGE 7 researchers themselves also highlight that their results could be different with agents other than oxaliplatin. Several non-randomized studies with conflicting results have been published comparing oxaliplatin-HIPEC with MMC-HIPEC [47–53]. In any case, published studies are retrospective and no meaningful comparison between the two drugs can be made regarding DFS and OS [54].

Given the lack of standardization of HIPEC, PSOGI and RENAPE groups have launched in November 2021 an ambitious project to formulate recommendations and guidelines for the use of HIPEC, based on a Delphi Consensus process of 140 international experts from 90 centres, trying to identify a HIPEC protocol to be evaluated in further clinical trials with the highest possible agreement. It seems that the preferred HIPEC regimen for PM-CRC is the one based on high-dose MMC (35 mg/m^2^), which will be the one used in our trial.

### Expected impact

This clinical trial, being multicenter, independent and randomized, acquires by itself a high value and impact in the scientific community. It is not usual to carry out clinical trials of this magnitude independently of the industry, and even less in the surgical field, which provides a superadded value due to the complete absence of commercial interest in the results. The results of this study can radically modify the treatment for these patients, since being a phase IV clinical trial, its results are directly applicable to clinical practice. Any advance in this area will directly benefit patients themselves in terms of cancer survival, and may entail financial savings for the health system by avoiding additional lines of high-cost chemotherapy, palliative interventions, and repeated hospital admissions. It is estimated that publications can be accepted in high-impact or first decile oncology and surgical journals.

## Data Availability

Data sharing not applicable to this article as no datasets were generated or analysed during the writing of the current study protocol.

## Abbreviations

AEMPS: *Agencia Española de Medicamentos y Productos Sanitarios*
CCS: Completeness of Cytoreduction Score
CRC: Colorectal cancer
CREC: Clinical Research and Ethics Committee
CRO: Clinical Research Organization
CRS: CytoReductive Surgery
CTCAE: Common Terminology Criteria for Adverse Events
DFS: Disease Free Survival
eCRF: Electronic Case Report Form
EORTC: European Organisation for Research and Treatment of Cancer
HIPEC: Hyperthermic IntraPEritoneal Chemotherapy
GECOP: *Grupo Español de Cirugía Oncológica Peritoneal*
MMC: Mytomicin-C
MTB: Multidisciplinary Tumor Board
OS: Overall Survival
PM: Peritoneal Metastases
PCI: Peritoneal Cancer Index
PSOGI: Peritoneal Surface Oncology Group International
RCT: Randomized clinical trial
RENAPE: *Réseau National de prise en charge des Tumeurs Rares du Péritoine*
RFS: Recurrence Free Survival
SCT: Systemic ChemoTherapy
UICEC: *Unidad de Investigación Clínica y Ensayos Clínicos*
